# Integrating knowledge graphs and retrieval-augmented generation to identify clinical pharmacology gaps in infectious disease trial protocols

**DOI:** 10.3389/fphar.2026.1955011

**Published:** 2026-10-14

**Authors:** Shreyas Waikar, Murali Ramanathan

**Affiliations:** Artificial Intelligence and Clinical Pharmacology Laboratory, Department of Pharmaceutical Sciences, University at Buffalo, The State University of New York, Buffalo, NY, United States

**Keywords:** artificial intelligence, clinical trials, global health, HIV, malaria, RAG, tuberculosis

## Abstract

**Purpose:**

Clinical trial protocols contain critical clinical pharmacology information that is often scattered across different sections of these lengthy documents. The purpose was to evaluate whether a retrieval-augmented generation (RAG) protocol review integrated with knowledge graphs (KGs) could be used to obtain clinical pharmacology evidence and identify regulatory gaps.

**Methods:**

Clinical trial protocols were obtained from the Gates Foundation’s publicly available corpus of clinical trials in low- and middle-income countries. The RAG protocol review was used to assess the protocols against Food & Drug Administration (FDA) guidance documents for hepatic impairment, exposure-response, population PK (PopPK), and physiologically based pharmacokinetic modeling (PBPK). Disease-specific KG analyses were conducted for tuberculosis (TB), human immunodeficiency virus (HIV), malaria, and neglected tropical diseases (NTD). KG models connecting studies to drugs, diseases, pharmacologic classes, organ-function exclusions, biomarkers, and pharmacokinetic (PK) parameter features were obtained.

**Results:**

Across 42 unique trials, the KG contained 223 nodes and 376 edges. TB protocols produced the largest disease-specific graph, whereas HIV had the highest frequency of PK studies. Gap analysis showed that hepatic impairment exclusions in TB were more frequent than PK studies. In the multi-guidance evaluation, faithfulness was high, answer relevancy was lower, and the clinical pharmacology metric showed guidance-dependent differences.

**Conclusion:**

The integrated KG-RAG approach can help highlight potential gaps in clinical pharmacology reporting in infectious disease clinical trial protocols.

## Introduction

Clinical trial protocols are central regulatory and scientific documents that define the study population, investigational treatment, dose selection, endpoint strategy, statistical analysis plan, and safety monitoring framework (Shin et al., 2026). The clinical pharmacology information needed to inform dosing decisions includes pharmacokinetic (PK) and pharmacodynamic (PD) assessments, biomarkers, exposure-response analyses, and eligibility criteria for hepatic and renal function. However, this information is frequently scattered across multiple protocol sections, embedded tables, eligibility criteria, appendices, and endpoint descriptions, making systematic comparison across trials difficult.

Infectious disease trials present a particularly relevant setting for structured protocol evaluation. Trials in tuberculosis (TB), human immunodeficiency virus (HIV), malaria, and neglected tropical diseases (NTDs) often involve complex treatment regimens, disease-specific biomarkers, heterogeneous patient populations, and organ impairment considerations (Ernest et al., 2021; McFeely et al., 2019; Rayner et al., 2021; Verrest et al., 2017). The clinical pharmacology components of a trial address dose selection and optimization, and patient-specific intrinsic and extrinsic factors such as age, gender, organ function, diet, and co-administered drugs (Minichmayr et al., 2024; Kaspera and Shitara, 2024; United States Food and Drug Administration FDA, 2022). Clinical pharmacology considerations are directly reflected in the choice of dosing regimen and implicitly in study design features such as inclusion-exclusion criteria and the timing of outcome assessment procedures (United States Food and Drug Administration FDA, 2022; Hrobjartsson et al., 2025). Clinical pharmacology information in infectious disease clinical trial protocols are reported inconsistently, making it difficult to systematically identify gaps in PK, drug-drug interaction (DDI) monitoring, biomarkers, organ impairment, and special populations across trials (Hrobjartsson et al., 2025; Soliman et al., 2022; Yap et al., 2023; Zhang et al., 2020). For example, excluding patients with hepatic or renal impairment may limit the generalizability of trial results to populations in which such diseases are prevalent. These gaps are difficult to detect through manual review when comparing multiple trials across disease areas.

Knowledge graphs (KGs) have emerged as an important framework for integrating heterogeneous biomedical information in drug development and clinical research, especially for LLM-assisted reasoning over biomedical knowledge, as highlighted in several reviews (Budhdeo et al., 2023; Cui et al., 2025; Xu et al., 2025). KGs have been used to address the data sparsity challenges inherent in rare disease research by leveraging standardized ontologies and semantic relationships (Fei et al., 2026). In pharmacovigilance, KGs have been applied to identify adverse events and DDIs (Hauben et al., 2024). Chen et al. constructed the Clinical Trials Knowledge Graph (CTKG) to infer knowledge from 8210 trials in ClinicalTrials.gov (Chen et al., 2022). CTKG represents studies, drugs, and conditions as nodes and relations such as drugs used in studies as edges, and their embedding analysis showed that the graph can support drug repurposing and similarity search (Chen et al., 2022). In particular, KGs could provide a semantic framework for holistically integrating clinical pharmacology information relevant to pharmacokinetic (PK) and pharmacodynamic (PD) modeling, population pharmacokinetics, physiologically based pharmacokinetic (PBPK) modeling, and other mechanistic pharmacometric approaches. By explicitly representing relationships among drug properties, biological processes, physiological systems, patient characteristics, covariates, biomarkers, and PK/PD parameters, KGs could complement conventional pharmacometric models and facilitate more systematic integration of heterogeneous evidence within model-informed drug development (MIDD) (Bhat et al., 2025). This can reveal recurring protocol patterns, deficiencies and missing information, and disease-specific differences in clinical pharmacology reporting.

Retrieval-augmented generation (RAG) evaluation provides a complementary method for protocol review as it is well-suited for document-restricted review tasks (Advani et al., 2026; Waikar et al., 2026; Wu et al., 2025). Our group has previously used RAG-LLM systems to evaluate drug information against FDA clinical pharmacology guidance and to assess clinical trial protocols. The system retrieved regulatory evidence and used it to identify compliance and deficiencies. The protocol assessment included PK parameters and PK-related information in the statistical analysis plan (Waikar et al., 2026). We have also investigated the strengths and limitations of the RAG strategy for assessing the immunogenicity data for biologic drugs from the package insert (Advani et al., 2026). Proestel et al. evaluated RAG architectures for answering questions related to the content of FDA guidance documents (Proestel et al., 2025). The RAG for clinical trial protocol reviews retrieves relevant protocol and guidance context and uses it to generate an evaluation and recommendation. However, standard RAG metrics such as answer relevancy and faithfulness may not fully capture whether a response includes the clinical pharmacology detail needed for decision-making. A domain-specific clinical pharmacology evaluation metric can therefore provide additional sensitivity to omissions, contradictions, and weak regulatory interpretation.

The objective of this proof-of-concept study was to develop and evaluate a KG-RAG framework for characterizing infectious disease clinical trial protocols and identifying relevant clinical pharmacology gaps. The analysis combined multi-guidance RAG evaluation, structured KG extraction, graph statistics, and gap queries. The central hypothesis was that AI tools such as KG and RAG can systematically evaluate large collections of trial protocols to reveal what the trials report, whom they exclude, and what information is missing across disease areas and regulatory guidance documents.

## Methods

### Clinical trial protocol datasets

Clinical trial protocols were downloaded from the publicly available clinical trial protocol registry published by the Design, Analyze, Communicate (DAC) division of the Gates Foundation (https://dac-trials.org/resources/protocol-library/) (Design et al., 2025). The registry included randomized controlled trials conducted in low- and middle-income countries with full protocols available and study designs published between 2015 and 2024. The registry contains 99 studies, the majority of which were conducted in Africa. The most frequent disease areas represented in the registry were TB, HIV, malaria, and NTDs.

Studies involving vaccine, neonatal and maternal, and nutritional interventions were excluded from the current analysis. Two HIV documents (Robb ECHO and CAPRISA HIPSS) were also excluded from the KG analysis because they were observational studies. Pneumonia trials were excluded because only two trials remained after the other filters. For NTD protocols, multiple PDF documents corresponding to the same ClinicalTrials.gov identifier were consolidated into a single trial unit of analysis. Three country-specific DOLF IDA documents (NCT02899936) were treated as one trial, and two fexinidazole documents with the same NCT number but different age-group focus (NCT02184689) were likewise treated as one trial.

### Protocol document preprocessing

The pipeline operated on disease-organized trial folders, with subfolders for TB, HIV, malaria, and NTDs containing protocol files in portable document format (PDF). Figure 1 is a schematic of the workflow in the KG and RAG.

**FIGURE 1:**
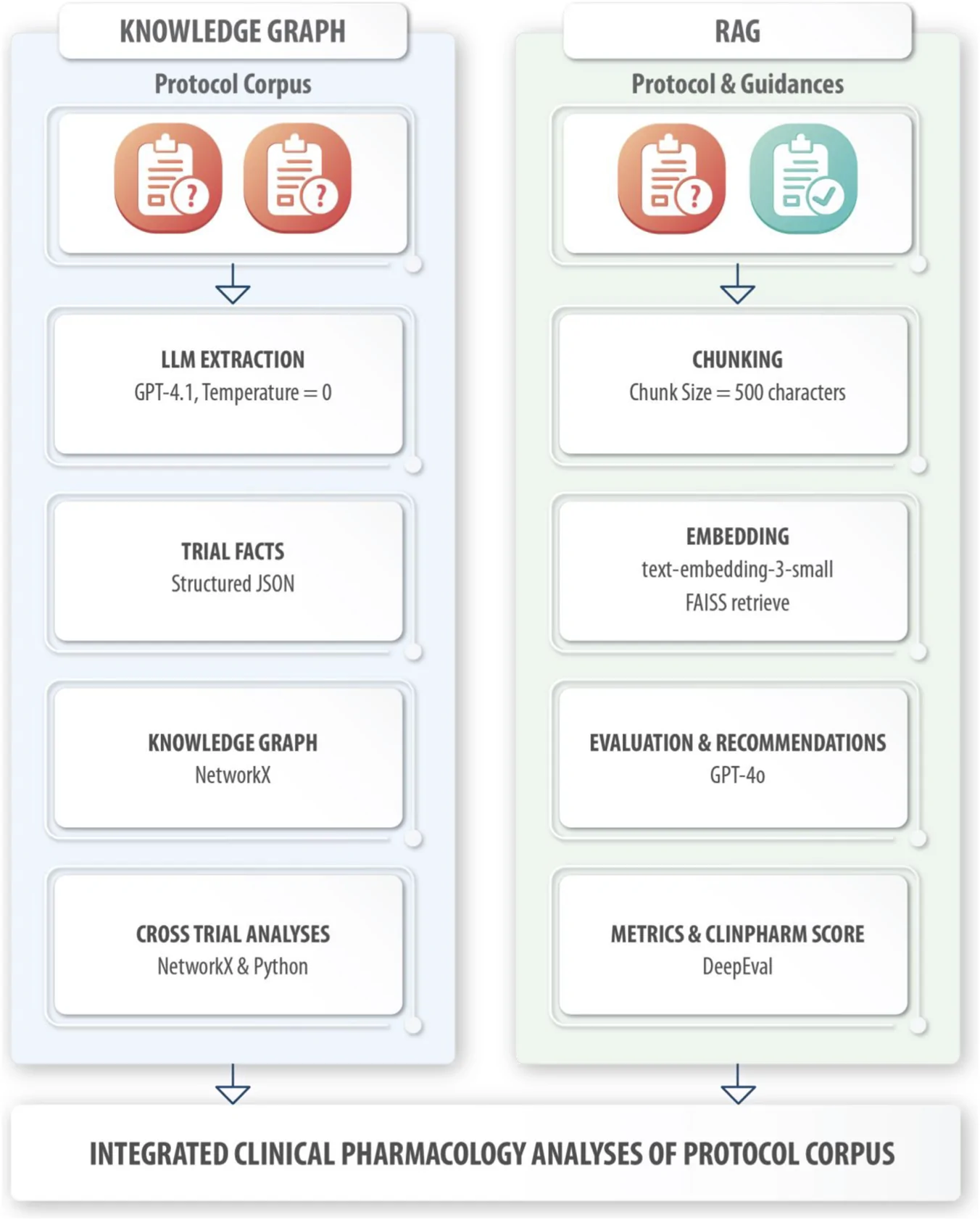
The workflow for the knowledge graph and retrieval-augmented generation analyses of clinical trial protocols. The goals of each step and the software packages used are indicated.

The unit of analysis was the individual clinical trial, identified by the National Clinical Trial (NCT) number, the 8-digit trial unique identifier assigned by the ClinicalTrials.gov database, and in some cases, an ISRCTN or ACTRN identifier. Each trial was represented in the KG as a trial node connected to extracted entities and protocol attributes. Disease-specific KG analyses were performed for TB, HIV, malaria, and NTDs.

Each PDF file was read using PyMuPDF for document loading, and whitespace normalization was applied by collapsing repeated newline characters into single spaces.

For RAG analysis, protocol and guidance documents were chunked with a recursive character splitter using a chunk size of 500 characters and zero overlap. The recursive splitter prioritized paragraph and whitespace boundaries before hard character cuts to better preserve local meaning. Tables embedded in the PDFs were extracted separately in markdown form and retained as distinct document objects to preserve structured pharmacology content that might otherwise be lost during plain-text parsing.

For protocol evaluation, inclusion, exclusion, and eligibility criteria were also identified using regular-expression section matching, with a fallback rule that captured pages containing those keywords when a contiguous eligibility section was not detected. For KG extraction, protocol text from each trial PDF was loaded in full and submitted to the extraction model. The identified inclusion, exclusion, and eligibility sections were used for extraction of enrollment criteria.

### Retrieval-augmented protocol evaluation

A RAG was used to evaluate protocol content against regulatory guidance. For each protocol PDF and guidance document, protocol chunks, extracted tables, and guidance document chunks were indexed in memory using OpenAI text-embedding-3-small embeddings and a FAISS vector database. Guidance context was retrieved with maximal marginal relevance search, and protocol context was retrieved by similarity search. An evaluation and recommendation were then generated with GPT-4o (temperature = 0). The prompts used are presented in Supplementary Material *LLM_prompts.docx*. The entire protocol and guidance document set was chunked, embedded, and indexed.

Each protocol was evaluated against four FDA guidance documents: Pharmacokinetics in Patients with Impaired Hepatic Function, Exposure-Response Relationships, Population Pharmacokinetics (United States Food & Drug Administration FDA, 2022), and Physiologically Based Pharmacokinetic Analyses.

The evaluation outputs were written as JSON files for each protocol-guidance pair and included the generated evaluation, recommendation, and associated DeepEval metrics for answer relevancy, faithfulness, and a clinical pharmacology score (DeepEval, 2025a; Ip and Vongthongsri, 2026).

#### Evaluation metrics and ClinPharm score

The RAG used DeepEval metrics to assess generated outputs (Ip and Vongthongsri, 2026). Scores were returned on a 0–1 scale and reported as percentages.

Answer relevancy measures how well the generated output addresses the input query. It was calculated with DeepEval’s Answer Relevancy metric using a gpt-3.5-turbo judge. Higher scores indicate that the response stayed on the question asked (DeepEval, 2025a).

Faithfulness measures whether claims in the generated output are supported by the retrieved protocol and guidance context. It was calculated with DeepEval’s Faithfulness metric using a gpt-4o judge (DeepEval, 2025b). Higher scores indicate that the response was grounded in the retrieved text rather than unsupported statements.

The ClinPharm score is a custom DeepEval GEval metric evaluated with gpt-4o. The ClinPharm score evaluated each generated output against three criteria (Shin et al., 2026): whether statements contradicted clinical pharmacology principles (Ernest et al., 2021), whether important details were omitted, and (McFeely et al., 2019) whether the content was relevant to drug development.

### KG analysis

#### LLM extraction of structured clinical trial facts

Structured trial-level facts were extracted for each clinical trial using a fixed JSON schema and then used as the input for KG construction. The schema included trial identifiers and disease labels, together with clinical pharmacology and protocol design fields such as drug name, therapeutic class, disease, phase, sample size, study design, hepatic-related exclusion criteria, renal-related exclusion criteria, pharmacokinetic (PK) study presence, exposure-response analysis, PK parameters, PK/pharmacodynamic endpoints, dose-response modeling, population PK (PopPK), therapeutic drug monitoring (TDM), biomarkers, and primary and secondary endpoints.

Extraction was performed using a deterministic prompting setup with GPT-4.1, with “*temperature*” set to 0, using the full protocol text. Binary protocol features such as hepatic exclusion, PK study inclusion, PopPK analysis, and exposure-response analysis were assigned by the model according to the prompt-defined schema and then parsed from the returned JSON. Invalid JSON outputs were recorded as parse errors.

#### KG construction

The extracted JSON facts were then converted into graph nodes and edges. The KG was constructed in Python using a directed NetworkX graph (28). Each trial was represented as a trial node, and additional nodes were created for drugs, diseases, biomarkers, PK parameters, therapeutic classes, pharmacologic classes, conditions, and study components as available from the extracted schema. Pharmacologic class (EPC) nodes were added separately by matching drug names to the FDA Products database. Directed edges were used to encode explicit relationships extracted from the trial facts, including *studies_drug*, *has_therapeutic_class*, *targets_disease*, *excludes*, *includes*, *measures*, and *uses_biomarker*. Exclusion edges connected trial nodes to hepatic or renal impairment nodes and could retain the corresponding exclusion text as an edge property. Shared study-component nodes were used for recurrent protocol features such as PK study, exposure-response analysis, dose-response modeling, PopPK, and TDM.

The resulting graphs were written to GraphML for downstream analysis, together with per-disease summary CSV and JSON artifacts containing all extracted facts.

#### KG characterization

Disease-specific KG characterization was performed using downstream statistical scripts operating on GraphML files. For each disease graph, the total numbers of nodes and edges were computed, together with node-type counts, edge-relation counts, and trial-level feature summaries derived from shared study-component and condition nodes. Trial-level protocol features were summarized by identifying whether each trial was connected to shared nodes representing PK study, PopPK, exposure-response analysis, hepatic exclusion, renal exclusion, and other extracted study components. Additional summaries included top-ranked entities based on number of linked trials, biomarker and PK parameter frequency, pharmacologic class distributions, and disease-level cross-comparison tables exported as CSV files for manuscript tables and plots.

Gap analysis was performed using Python and NetworkX query functions (Hagberg et al., 2008). The queries evaluated patterns such as trials that excluded patients with hepatic or renal impairment but lacked an explicit PK study, shared biomarkers across trials, and disease-level comparisons of exclusion criteria and protocol features. These analyses were implemented as reusable query functions over the GraphML representation and were designed to identify regulatory or pharmacology-relevant gaps exposed by the graph structure. Gap-analysis results were generated as query reports from the GraphML representation.

### Software implementation and statistical analyses

#### Software implementation

The overall implementation was in Python and used LangChain (2023), LangChain OpenAI interfaces, FAISS, PyMuPDF, pandas, DeepEval, and related utility packages. The KG analysis used NetworkX and GraphML (Hagberg et al., 2008).

The pipeline generated multiple outputs. The RAG analysis wrote one timestamped JSON file per trial document per guidance-specific output folder. The KG analysis wrote NCT-level facts JSON files as well as disease-level GraphML, summary CSV, and aggregated all-facts JSON files. The statistics scripts exported disease-level and cross-disease summary CSVs, and scripts generated additional wide-format CSV outputs for comparative analysis.

#### Statistical analysis

Statistical analyses were conducted with *rstatix* and other R packages (Kassambara, 2023; R Core Team, 2017). We used three-way ANOVA with the formula Score ∼ Metric*DiseaseArea*Guidance to compare scores in Figure 2 across Answer Relevancy, Faithfulness, and ClinPharm metrics, disease area factor and guidance. We used one-way ANOVA to compare Answer Relevancy, Faithfulness, and ClinPharm metrics in Figure 3 across the disease area factor. Effect sizes were assessed with generalized eta-square (GES) values. Pairwise *post hoc* comparisons used the Games-Howell test.

**FIGURE 2:**
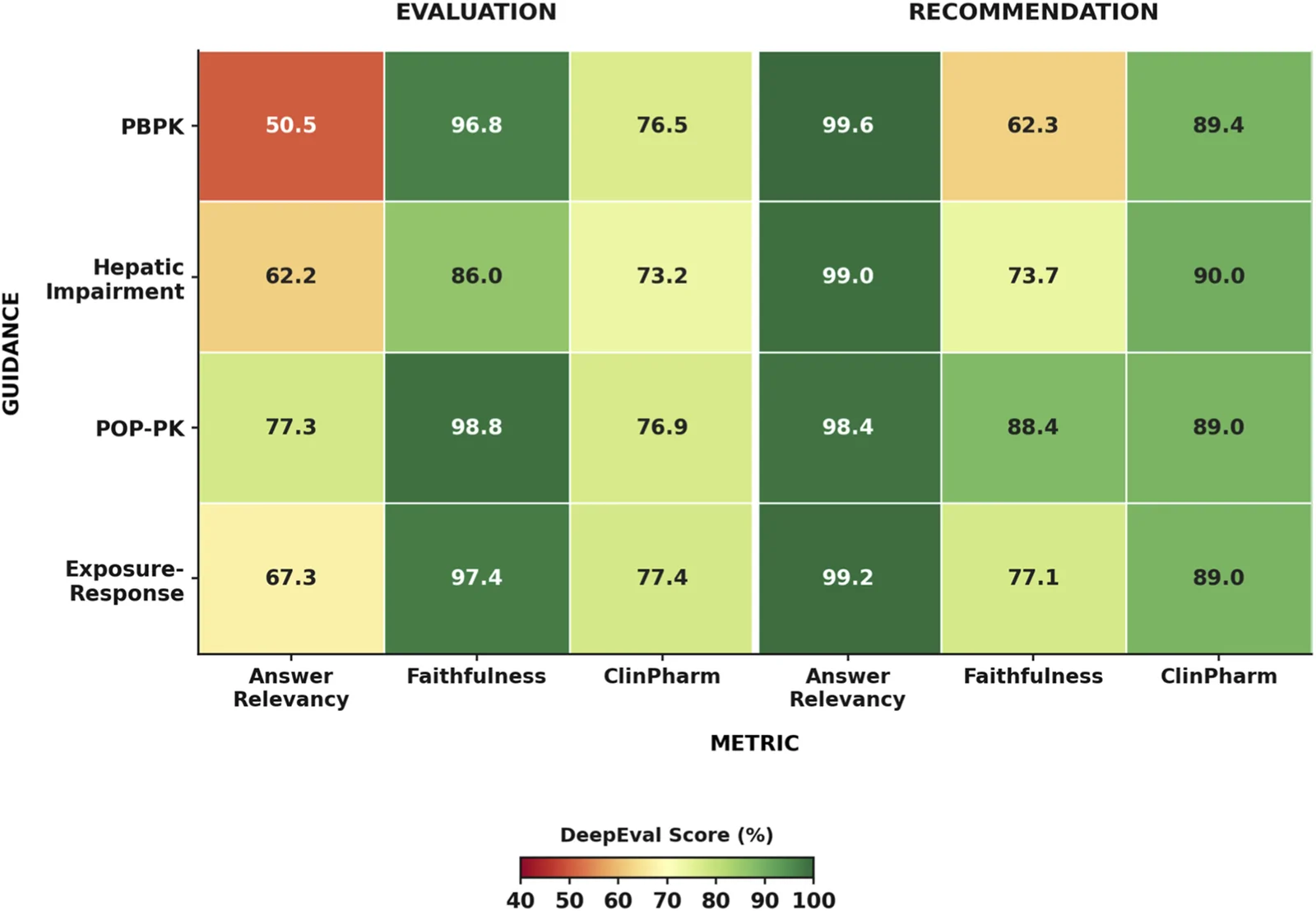
Mean DeepEval scores across every guidance, phase, and metric. The heatmap compares answer relevancy, faithfulness, and ClinPharm scores during the evaluation and recommendation phases.

**FIGURE 3:**
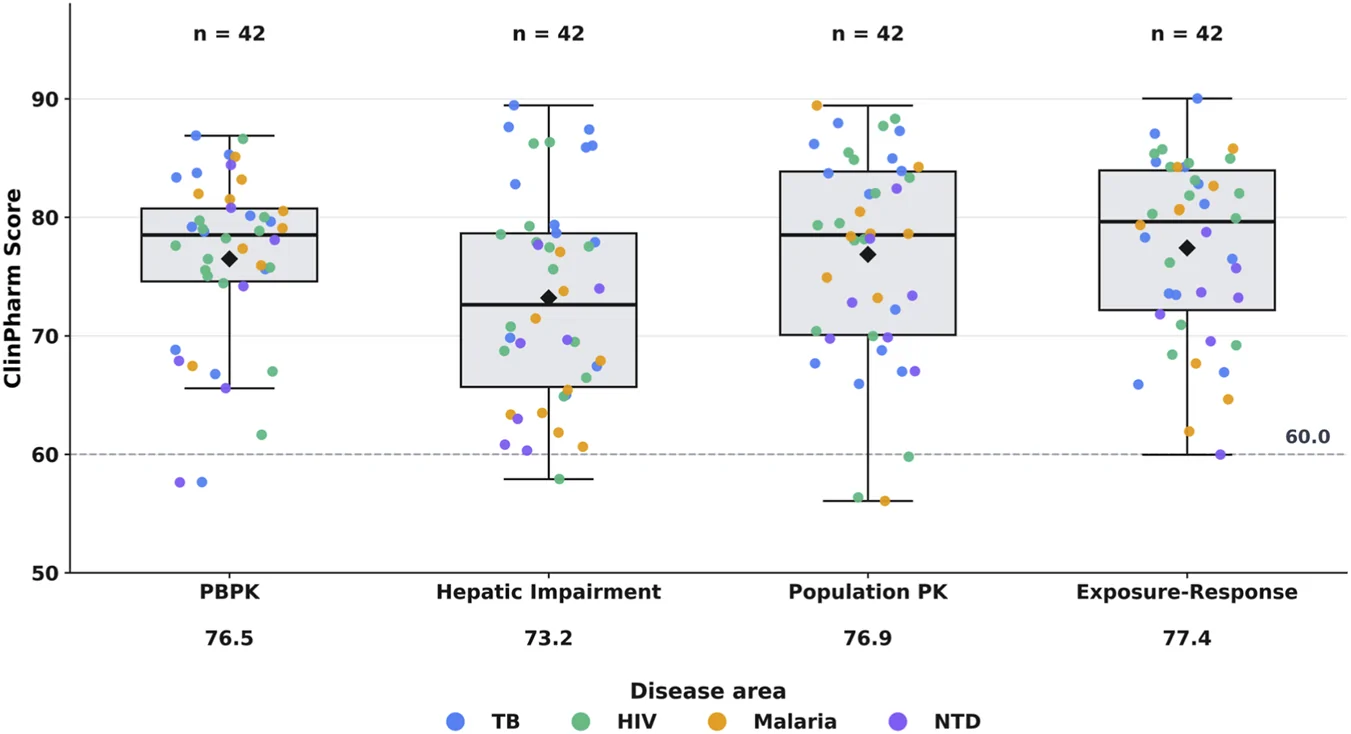
The boxplots and overlaid trial-level points show the distribution of clinical pharmacology scores for the protocol evaluations (*y*-axis) across PBPK, Hepatic Impairment, Population PK, and Exposure-Response regulatory guidance (*x*-axis). The black diamond and the number below each box represent the mean. The horizontal line inside each box is the median, and the sample size is shown above each box. The filled circles correspond to the individual protocol evaluations for each disease area, as shown in the legend.

## Results

### KG characterization of the LMIC clinical trial dataset

#### Salient features

Structured information was extracted from 42 clinical trial protocols, comprising 12 TB, 14 HIV trials, 9 malaria, and 7 NTD trials. Supplementary Table S1. *docx* summarizes the NCT numbers, disease areas, drugs, and the countries in which the trials were conducted.

The combined cross-disease KG contained 223 nodes and 376 edges (Figure 4). In Figure 4, the trial nodes are connected to disease areas and drug nodes. The trial-disease and trial-drug connections for the KG are provided as a CSV file in Supplementary Material *kg.csv*. The number of nodes and edges quantifies graph size and the number of edges per node is the measure of the connectivity.

**FIGURE 4:**
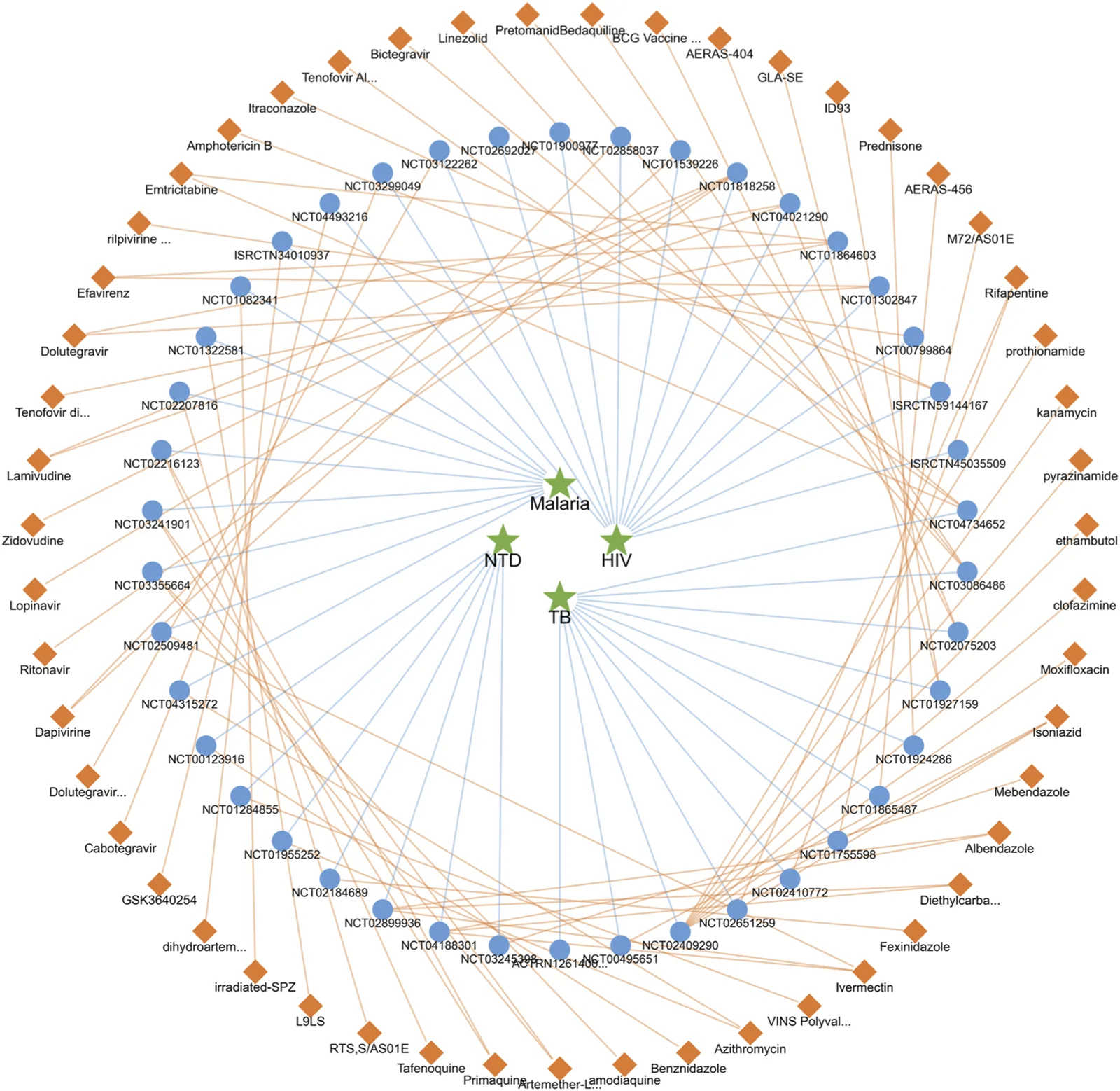
Knowledge graph representation of the clinical trial protocols. The network diagram shows trial nodes connected to diseases and drugs. Abbreviations: HIV: Human immunodeficiency virus; NTD: Neglected tropical diseases; TB: Tuberculosis.

The disease-specific graphs differed in size and density. The TB KG contained 93 nodes and 134 edges, the HIV KG contained 75 nodes and 129 edges, the malaria KG contained 51 nodes and 62 edges, and the NTD KG contained 35 nodes and 50 edges. TB produced the largest disease-specific graph (93 nodes and 134 edges), whereas HIV had the highest PK study rate (64%). Malaria ranked third in graph size (51 nodes and 62 edges) with a PK study rate of 44.4%.

#### Drug representation

Across the KG, 13 drugs appeared in two or more trials, and the remaining 38 drug entities were singletons. Figure 5 shows the top-ranked drugs by trial frequency: isoniazid and ivermectin were the most frequent (3 trials each), followed by rifapentine, dolutegravir, artemether-lumefantrine, primaquine, efavirenz, lamivudine, albendazole, and diethylcarbamazine (2 trials each). This distribution indicates repeated investigation of a small set of interventions within a broader heterogeneous protocol corpus.

**FIGURE 5:**
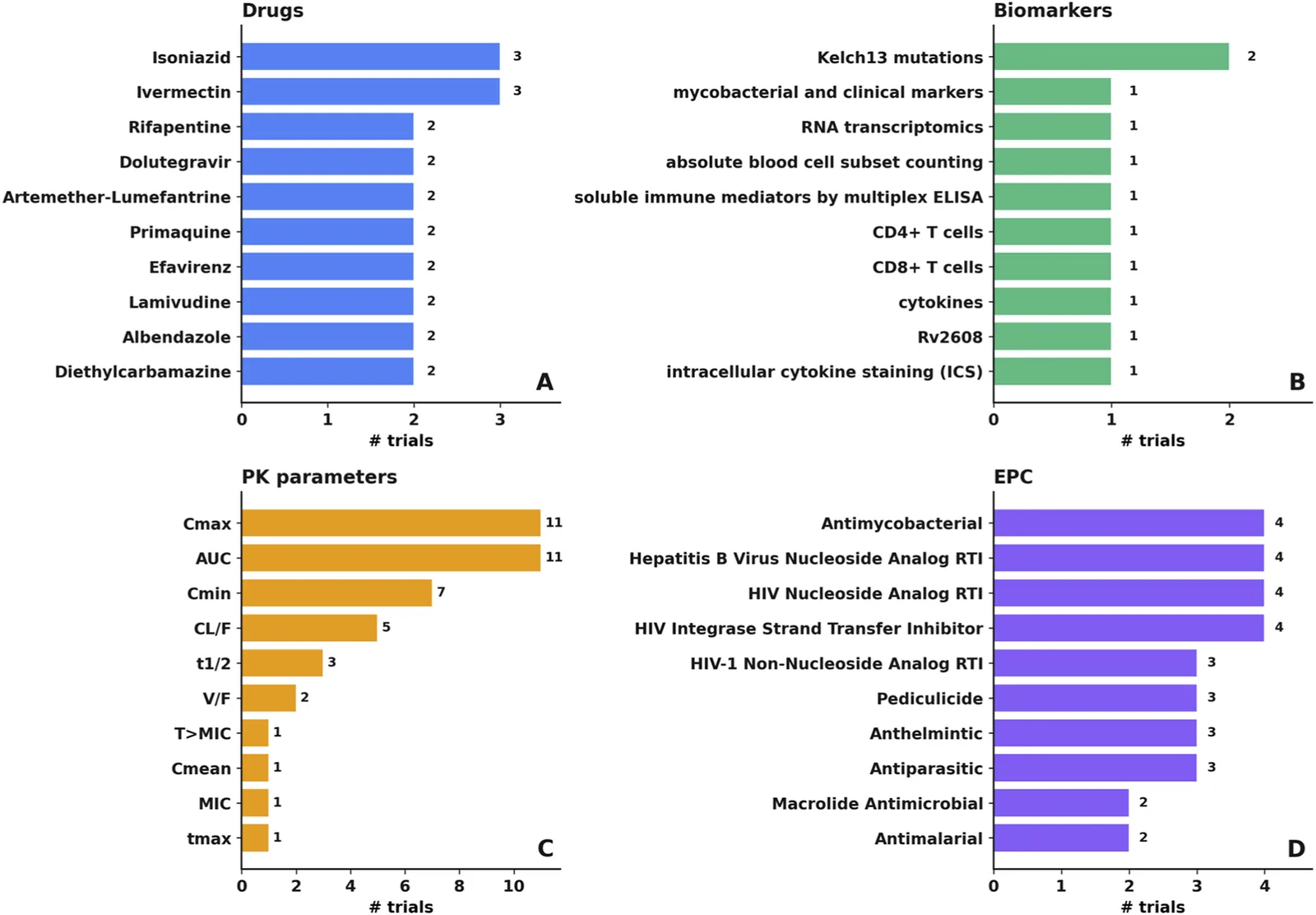
Most frequently extracted entities in the knowledge graph. **(A–D)** Show the top drugs, biomarkers, PK parameters, and FDA-established pharmacologic class (EPC) labels by number of trials. Abbreviations: AUC: Area under the curve; HIV: Human immunodeficiency virus; RTI: Reverse transcriptase inhibitor; t1/2: Half-life; T > MIC: Time concentration is greater than minimum inhibitory concentration.

Biomarker utilization was comparatively sparse. Kelch13 mutations were the only biomarker linked to more than one trial, whereas other immune, mycobacterial, and cellular markers appeared once (Figure 5B). As expected, noncompartmental PK parameters such as Cmax and AUC were most frequent (11 trials each), followed by Cmin, CL/F, and t1/2, whereas tmax appeared less often (Figure 5C). Antimycobacterial agents and HIV nucleoside analog RTIs, hepatitis B virus nucleoside analog RTIs, and HIV integrase strand transfer inhibitors were the most frequent EPC classes (Figure 5D).

#### Clinical pharmacology specific patterns

Clinical pharmacology features also differed across diseases. Figure 6 summarizes cross-disease, KG-derived protocol features across clinical pharmacology considerations, patient exclusions, and information gaps.

**FIGURE 6:**
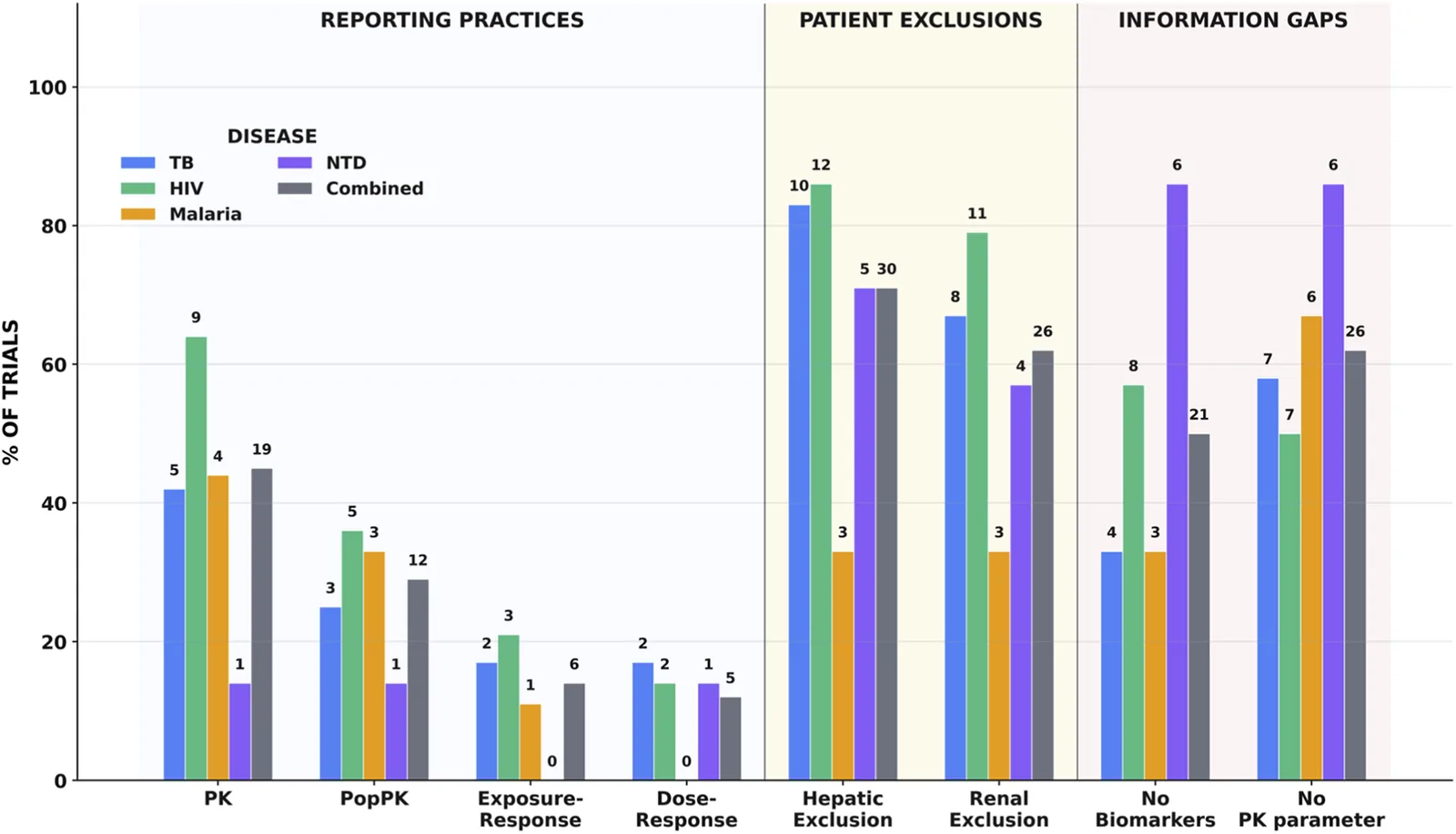
Knowledge graph compliance profile by disease. The grouped bar chart summarizes reporting practices, patient exclusions, and information gaps across tuberculosis (TB), HIV, malaria, NTD, and the combined dataset. The number over each bar is the count.

Across the 42 trials, 45% included a PK study, 29% included a PopPK analysis, and 14% included an exposure-response analysis (Figure 6). HIV protocols (64%) most frequently contained PK studies, followed by malaria (44%), TB (42%), and NTD (14%). PopPK analysis was less frequent, appearing in 36% of HIV trials, 33% of malaria trials, 25% of TB trials, 14% of NTD trials. Exposure-response analysis was infrequent, occurring in 21% of HIV trials, 17% of TB trials, 11% of malaria trials, and 0% of NTD trials.

Hepatic impairment exclusions were common, especially in HIV and TB trials, and were also frequent in the combined cohort. Renal exclusions were less frequent but occurred in every disease area. In the TB trials, hepatic impairment exclusion criteria were present in 83% of trials; however, only 42% included a PK study. Thus, protocols often restricted enrollment based on organ function, while the downstream PK, PopPK, exposure-response, or dose-response analyses that could inform dosing in these excluded populations were less consistently represented.

Biomarker reporting was limited in NTD protocols. No therapeutic drug monitoring (TDM) features were identified. Because TDM-related language may be represented heterogeneously across protocols, this finding should be interpreted in the context of the extraction schema.

Taken together, the KG analysis identified differences among TB, HIV, malaria, and NTD protocols in graph size and clinical pharmacology content. The extracted structure also exposed areas of limited coverage, particularly for PopPK, exposure-response analysis, and TDM-related features.

### Multi-guidance evaluations and ClinPharm scores

Figure 2 summarizes DeepEval Answer Relevancy, Faithfulness, and ClinPharm scores across guidance areas for clinical trial protocol evaluations and recommendations. Figure 3 summarizes trial-level ClinPharm score distributions by guidance.

In the ANOVA analyses of the DeepEval scores for protocol evaluations, we found significance on the Metric (*p* < 0.001, GES = 0.387), Disease (*p* < 0.001, GES = 0.045), Guidance (*p* < 0.001, GES = 0.071), and the Metric:Disease two-way interaction (*p* < 0.001, GES = 0.080). The mean Faithfulness score was high (94.6%), whereas the mean Answer Relevancy was lower (64.3%), and ClinPharm was intermediate (76.0%).

In the ANOVA analyses of the Deepeval scores for protocol recommendations, we found significance on the Metric (*p* < 0.001, GES = 0.235), Guidance (*p* = 0.006, GES = 0.027), and the Metric:Guidance two-way interaction (*p* < 0.001, GES = 0.063). Recommendation Answer Relevancy was the highest (99.1%), while Recommendation Faithfulness was lower (75.1%). Hepatic impairment evaluations had the lowest median ClinPharm score (71.1%), whereas Exposure-Response had the highest (79.0%).

The scatter-boxplots in Figure 3 summarize the distributions of ClinPharm metric for protocol evaluation relative to the PBPK, Hepatic, PopPK, and Exposure-Response guidance documents. Exposure-Response produced the highest median ClinPharm score, whereas hepatic impairment produced the lowest; PBPK and PopPK were intermediate and exhibited trial-to-trial variability.

In the ANOVA analysis of ClinPharm scores for protocol evaluations, we found differences between disease areas relative to the hepatic impairment guidance (one-way ANOVA, *p* = 0.002, GES = 0.329). We did not obtain evidence that ClinPharm scores varied by disease area for the other guidance documents (all *p* > 0.05). In *post hoc* pairwise Games-Howell tests, significant differences in ClinPharm scores on the hepatic impairment guidance were found for the Malaria vs. TB (*p*_*adjusted*_ = 0.0033, Estimate = 12.6, 95% CI = 3.89–21.2) and NTD vs. TB (*p*_*adjusted*_ = 0.018, Estimate = 11.9, 95% CI = 1.86–22.0) comparisons.

There was substantial trial-to-trial variability, and individual protocols could have focal gaps. ClinPharm scores, which provide a summative score across multiple clinical domains, should be considered alongside guidance-specific regulatory gap detection measures to ensure the effectiveness of the review process.

## Discussion

This proof-of-concept study demonstrates the feasibility of using KG analysis to convert infectious disease clinical trial protocols into a structured representation of their clinical pharmacology features. The KG representation preserved trial-level relationships among diseases, drugs, pharmacologic classes, exclusions, biomarkers, PK parameters, and study components. This structure enabled cohort-level analysis that would be difficult to perform reliably through manual protocol review alone.

A potential limitation is that the study includes only 42 trials, which could limit the robustness of the inferential claims. The research was deliberately focused on global infectious disease trials with drug interventions from the Gates Foundation library rather than on a broader repository such as ClinicalTrials.gov. This targeted approach enhances the relevance and potential impact of the findings in low- and middle-income countries (LMICs), where infectious diseases remain a substantial public health burden. However, it may limit generalizability to disease and treatment settings, particularly those prevalent in high-income countries. Additional validation will be important to establish the robustness and generalizability.

The broader literature has demonstrated substantial heterogeneity and incompleteness in clinical trial protocol reporting. The updated SPIRIT 2025 statement, which provides a 34-item checklist for randomized clinical trial protocols, noted substantial variation in protocol completeness and emphasized that protocols are critical for enabling scientific, ethical, safety, and operational oversight (Chan et al., 2025). A systematic review of randomized trial protocols found that complete reporting of monitoring scope, frequency, and organization was uncommon: only 8% of trials provided complete information, and 48% reported some information (Hsieh et al., 2023). Similarly, assessments of eligibility reporting have shown that important information relevant to trial applicability and external validity is frequently incompletely reported. (Miranda et al., 2024). Our findings extend this literature, which has generally focused on methodological domains such as eligibility, outcomes, monitoring, randomization, and statistical analysis, by examining protocols through a clinical pharmacology lens. Our clinical pharmacology analysis connects protocol characteristics to PK, PopPK, exposure-response, hepatic and renal impairment, biomarkers, and pharmacologic classes. This distinction is important because a protocol can satisfy conventional reporting requirements while still providing insufficient information to determine whether its dosing strategy adequately addresses exposure variability or clinically relevant special populations. The KG therefore provides a complementary perspective in which apparently separate protocol elements can be evaluated as interconnected clinical pharmacology relationships.

The KG analysis showed that TB produced the largest disease-specific graph, whereas HIV had the highest frequency of PK studies. TB protocols showed a different signal: hepatic impairment exclusions were frequent, but PK study representation was much lower. Isoniazid and ivermectin were the most frequently repeated drugs, while antimycobacterial and HIV-related EPC classes were among the most common pharmacologic-class entities, and AUC and Cmax were the most frequent PK parameters. This pattern is clinically important because excluding patients with impaired hepatic function can limit generalizability and may leave dosing uncertainty unresolved unless supported by dedicated PK, PopPK, or exposure-response evidence. However, it is noted that some infectious disease trials may prioritize clinical or microbiological endpoints and incorporate PK or PopPK characterization through separate studies or previously generated evidence rather than within the primary efficacy protocol.

Clinical pharmacology considerations, such as renal and hepatic failure, can warrant careful drug selection and dose adjustments for individual drugs. For example, HIV patients are at risk for kidney failure caused by HIV-associated nephropathy and have an increased risk of liver injury due to hepatitis B and C virus infections. In TB, pyrazinamide and isoniazid carry a greater risk of hepatotoxicity than rifampin (Dhiman et al., 2012), and chronic kidney disease is common in countries such as Bangladesh, where TB is prevalent (Kar et al., 2023). TB patients can have lower body weight than the general population. Across the 42 trials, PK studies were identified in 45% of protocols, whereas PopPK and exposure-response analyses were identified in only 29% and 14%, respectively. These approaches can be informative in infectious disease trials because treatment response and drug exposure can vary with body size, organ function, disease severity, concomitant medications, age, nutritional status, and other patient-level characteristics. Thus, the KG analysis identifies gaps that could represent an opportunity for a global infectious disease program. Because KGs capture the reporting architecture of the protocol corpus rather than the complete pharmacometric evidence base for each drug, the identification of individual gaps by KG should not be interpreted as evidence that the corresponding analyses were scientifically required or that they were not performed elsewhere.

In ANOVA plus *post hoc* pairwise Games-Howell tests, the ClinPharm scores on the hepatic impairment guidance differed for the Malaria vs. TB (95% CI = 3.89–21.2) and NTD vs. TB comparisons with similar effect sizes (Estimate = 12.6 and 11.9). Figure 3 reflects this: most TB ClinPharm scores are above the median, while most malaria and NTD ClinPharm scores are below the median. However, the underlying causes remain poorly understood. Across guidance documents, evaluation Faithfulness was high, whereas evaluation Answer Relevancy was lower, and ClinPharm was intermediate. Recommendation Answer Relevancy was highest, while recommendation Faithfulness was lower. Hepatic impairment evaluations had the lowest median ClinPharm score. This divergence suggests that standard LLM evaluation metrics may overestimate the quality of regulatory evaluations when the key weakness is omitting domain-specific clinical pharmacology detail rather than hallucination or topical irrelevance.

The contribution of the study is the use of KGs as a clinical pharmacology reasoning aid, rather than as a predictive model or biomedical knowledge repository, because it links trials to clinical pharmacology features, such as drugs, diseases, organ-function exclusions, and population PK. KGs can answer questions that are challenging to address from unstructured protocol text alone. KGs also complement RAGs, which are well-suited to protocol retrieval and evaluation (Shin et al., 2026; Waikar et al., 2026). The KG exposes structured relationships, reporting patterns, and gaps across the protocol corpus, whereas the RAG evaluates individual protocols against guidance-specific expectations and generates recommendations for improvement. A possible use case for KG-RAG implementation is as part of an AI-enabled, human-in-the-loop trial portfolio management system.

The KG depends on an LLM for extraction, and extracted entities may reflect both true protocol content and extraction errors. Some protocol content may be embedded in complex tables, figures, appendices, or scanned regions that are not fully captured by text extraction or simple table conversion. The RAG output was evaluated using automated metrics, including an LLM-as-a-judge clinical pharmacology score. In our RAG study, we manually checked drug-information findings against the source labels and manually compared protocol comments with FDA E9 guidance (Waikar et al., 2026). Automated scores are a useful tool for screening, but are not a substitute for expert review. Although this metric was designed to penalize omissions and regulatory weaknesses, it does not replace expert adjudication by clinical pharmacologists, statisticians, or regulatory reviewers. To build confidence in LLM evaluations of clinical trial protocols, validation against the gold standard of a panel of expert reviewers is essential. Given the interdisciplinary nature and substantial length of these documents, along with the potential for inter-rater variability, scaling expert panel evaluations across a large corpus of protocols will be challenging. One potential approach is to have expert panels independently verify LLM-generated evaluations and recommendations against the source documents. However, over time, the increasing adoption of LLMs by subject-matter experts, together with rigorous validation in analogous regulatory and compliance settings, may generate real-world evidence that supports or challenges LLM performance in clinical trial protocols.

In conclusion, our results provide proof of concept that KG integrated with RAG can systematically characterize infectious disease clinical trial protocols and highlight potential gaps in clinical pharmacology reporting. The approach identified disease-specific differences in graph structure, PK reporting, patient exclusions, biomarker extraction, and guidance alignment. However, these signals should be interpreted cautiously, as missing extracted features or lower guidance-alignment scores do not, by themselves, establish regulatory non-compliance. With additional validation, this RAG-KG framework may help sponsors and regulatory agencies prioritize protocols for human review of the completeness of clinical pharmacology.

## Data Availability

The original contributions presented in the study are included in the article/Supplementary Material, further inquiries can be directed to the corresponding author.
