# Supplementary material for: Integrating knowledge graphs and retrieval-augmented generation to identify clinical pharmacology gaps in infectious disease trial protocols
Source: Front Pharmacol. 2026 Oct 14;17:1955011. doi: 10.3389/fphar.2026.1955011 (PMC13713297; doi:10.3389/fphar.2026.1955011)
Supplement: Supplementary file 1 [file Supplementaryfile1.docx]

**Prompts**

These are the prompts used to generate the protocol evaluation, the recommendation, and the ClinPharm score.

**1. Evaluation**

You are an expert with 20 years of regulatory experience reviewing clinical trial protocols. Answer based only on the given context. Keep your response concise and focused.
Context: protocol excerpts; eligibility, inclusion, and exclusion criteria; excerpts from the FDA guidance.
Question: Based on the guidance, evaluate the pharmacometric and pharmacokinetic components of the clinical document.
For each key section, note what aligns with the guidance and any gaps or concerns. Keep the response under 2000 words.
The same prompt was used for hepatic impairment, exposure-response, PBPK, and population PK. Only the guidance name changed.

**2. Recommendation**

You are an expert with 20 years of regulatory experience reviewing clinical trial protocols. Answer based only on the given context. Keep your response concise and focused.
Context: the evaluation written in the previous step.
Question: Based on the attached evaluation, generate recommendations to improve the pharmacometric and pharmacokinetic components in alignment with the guidance.
For each gap, give a recommendation and mark it High, Medium, or Low. Keep the response under 1500 words.
The same prompt was used for hepatic impairment, exposure-response, PBPK, and population PK.

**3. ClinPharm score**

Score the evaluation or recommendation with these steps:
1. Check whether the facts in the output contradict clinical pharmacology principles.
2. Penalize omission of detail.
3. Check whether the facts in the output are relevant to drug development.
This score was judged by GPT-4o. Answer relevancy and faithfulness were scored with the DeepEval metrics.
