# Supplementary material for: Integrating knowledge graphs and retrieval-augmented generation to identify clinical pharmacology gaps in infectious disease trial protocols
Source: Front Pharmacol. 2026 Oct 14;17:1955011. doi: 10.3389/fphar.2026.1955011 (PMC13713297; doi:10.3389/fphar.2026.1955011)
Supplement: Supplementary file 2 [file Table1.docx]

**Supplementary Table S1.** Identifiers, countries, and protocol descriptions of the included trials.

| **Trial ID (countries)** | **Trial title and brief description** |
| --- | --- |
| **HIV (*n* = 14)** | |
| ISRCTN45035509  **Countries:** Cameroon, Malawi, Tanzania, Zambia | **Title:** A Phase III, Randomised, Controlled Trial for the Treatment of HIV-Associated Cryptococcal Meningitis: Oral Fluconazole Plus Flucytosine or One Week Amphotericin B-Based Therapy vs Two Weeks Amphotericin B-Based Therapy  **Brief description:** drug(s)/intervention(s): Antifungal combinations. |
| ISRCTN59144167  **Countries:** Vietnam | **Title:** A Randomized, Open-Label, Comparative Study of the Effectiveness of Itraconazole versus Amphotericin B in the Induction Treatment of Penicilliosis in HIV-Infected Adults (IVAP)  **Brief description:** drug(s)/intervention(s): Itraconazole; Amphotericin B; design/arms: open-label, non-inferiority, randomized. |
| NCT00799864  **Countries:** India, Kenya, Romania, South Africa, Thailand, Uganda, Ukraine, United States) | **Title:** A Phase II, open-label, single-arm trial to evaluate the pharmacokinetics, safety, tolerability, and antiviral activity of rilpivirine (TMC278) in antiretroviral-naïve HIV-1 infected adolescents and children aged ≥6 to <18 years.  **Brief description:** Phase 2; drug(s)/intervention(s): Rilpivirine; design/arms: open-label, single-arm. |
| NCT01302847  **Countries:** Botswana, Brazil, Kenya, South Africa, Tanzania, Thailand, United States, Zimbabwe | **Title:** Phase III Study of the Virologic Efficacy and Safety of Dolutegravir-Containing versus Efavirenz-Containing Antiretroviral Therapy Regimens in HIV-1-Infected Pregnant Women and their Infants.  **Brief description:** Phase 3; drug(s)/intervention(s): Dolutegravir; Efavirenz; Antiretroviral Therapy; Arm 1: Maternal dolutegravir plus emtricitabine/tenofovir al; design/arms: non-inferiority. |
| NCT01539226  **Countries:** South Africa, Uganda | **Title:** A Multi-Centre, Randomised, Double-Blind, Placebo-Controlled Phase II Long-Term Safety Trial of a Dapivirine Vaginal Matrix Ring in Healthy HIV-Negative Women.  **Brief description:** Phase 2; drug(s)/intervention(s): Dapivirine; design/arms: double-blind, placebo-controlled, randomized. |
| NCT01818258  **Countries:** Malawi, Tanzania, Uganda, Zimbabwe | **Title:** Phase IV Evaluation of the Steady State Pharmacokinetics of Zidovudine, Lamivudine, and Lopinavir/Ritonavir in Severely Malnourished HIV-1-Infected Children.  **Brief description:** Phase 4; drug(s)/intervention(s): Zidovudine; Lamivudine; Lopinavir/Ritonavir. |
| NCT01864603  **Countries:** Kenya, Uganda | **Title:** Sustainable East Africa Research in Community Health (SEARCH)  **Brief description:** design/arms: cluster-randomized, randomized. |
| NCT01900977  **Countries:** South Africa, Zambia | **Title:** Population Effects of Antiretroviral Therapy to Reduce HIV Transmission (PopART): A Cluster-Randomized Trial of the Impact of a Combination Prevention Package on Population-Level HIV Incidence in Zambia and South Africa  **Brief description:** drug(s)/intervention(s): Antiretroviral Therapy; design/arms: cluster-randomized, randomized. |
| NCT02692027  **Countries:** Lesotho | **Title:** Same Day Community-Based ART Initiation Versus Clinic-Based Pre-ART Assessment and Counselling for Individuals Newly Tested HIV-Positive During Community-Based HIV Testing in Rural Lesotho – a Randomized Study  **Brief description:** design/arms: open-label, cluster-randomized, randomized. |
| NCT02858037  **Countries:** Malawi, South Africa | **Title:** A Phase 3B Open-Label Follow-on Trial to Assess the Continued Safety of and Adherence to a Vaginal Ring Containing Dapivirine in Women  **Brief description:** Phase 3B; drug(s)/intervention(s): Dapivirine; design/arms: open-label. |
| NCT03299049  **Countries:** Argentina, Australia, Canada, France, Germany, Italy, Mexico, Russia, South Africa, South Korea, Spain, Sweden, United States | **Title:** A Phase 3 Double Blind Safety and Efficacy Study of Long-Acting Injectable Cabotegravir Compared to Daily Oral TDF/FTC for Pre-Exposure Prophylaxis in HIV-Uninfected Women  **Brief description:** Phase 3; drug(s)/intervention(s): Cabotegravir; design/arms: open-label, double-blind, non-inferiority, randomized. |
| NCT04021290  **Countries:** Argentina, Belgium, Canada, China, Denmark, France, Germany, Italy, Mexico, Russia, South Africa, Spain, Sweden, Taiwan, United Kingdom, United States | **Title:** A Phase III, Randomized, Multicenter, Open-Label, Non-Inferiority Study Evaluating the Efficacy, Safety and Tolerability of Switching to Dolutegravir/Lamivudine Fixed-Dose Combination in HIV-1 Infected Adults Who Are Virologically Suppressed  **Brief description:** Phase 3; drug(s)/intervention(s): Dolutegravir/lamivudine; design/arms: open-label, non-inferiority, randomized. |
| NCT04493216  **Countries:** Argentina, Canada, France, Germany, Italy, Portugal, Russia, South Africa, Spain, Switzerland, United States | **Title:** A Randomized, Double-Blind (Sponsor-unblinded), Placebo-Controlled, Adaptive Trial to Investigate the Antiviral Effect, Safety, Tolerability and Pharmacokinetics of GSK3640254 in HIV-1 Infected Treatment-Naïve Adults  **Brief description:** Phase 2A; design/arms: double-blind, placebo-controlled, randomized. |
| NCT03122262  **Countries:** South Africa | **Title:** A 96-week Randomised, Phase 3 Non-inferiority Study of DTG + TAF + FTC Compared with DTG + TDF + FTC and EFV + TDF + FTC in Patients Infected with HIV-1 Starting First-line Antiretroviral Therapy  Brief description: Phase 3; design/arms: open-label, non-inferiority, randomized. |
| **MALARIA (*n* = 9)** | |
| ISRCTN34010937  **Countries:** Indonesia | **Title:** Efficacy and Safety of Intermittent Preventive Treatment and Intermittent Screening and Treatment Versus Single Screening and Treatment with Dihydroartemisinin–Piperaquine for the Control of Malaria in Pregnancy in Indonesia: A Cluster-Randomised, Open-Label, Superiority Trial  **Brief description:** design/arms: open-label, cluster-randomized, randomized. |
| NCT01082341  **Countries:** Colombia | **Title:** Phase 1 and 2a Clinical Trial: Immunization of Human Volunteers with *P. vivax* Irradiated Sporozoites  **Brief description:** Phase 1. |
| NCT01322581  **Countries:** Mali | Title: Safety and Efficacy of L9LS, a Human Monoclonal Antibody Against *Plasmodium falciparum*, in a Dose-Escalation Trial in Adults and Children and a Randomized, Double-Blind Trial of Children in Mali  Brief description: Phase 2; design/arms: open-label, double-blind, randomized. |
| NCT02207816  **Countries:** Burkina Faso, Kenya, Tanzania | **Title:** An Open Extension to the Phase III, Multi-Center Study MALARIA-055 PRI (110021) to Evaluate Long-Term Efficacy, Safety and Immunogenicity of the RTS,S/AS01E Candidate Vaccine Against Malaria Disease Caused by *Plasmodium falciparum* in Infants and Children in Africa  **Brief description:** Phase 3; drug(s)/intervention(s): vaccine against; design/arms: randomized. |
| NCT02216123  **Countries:** Brazil, Colombia, Ethiopia, Peru, Thailand, Vietnam | **Title:** A Randomized, Double-Blind, Double Dummy, Comparative, Multicenter Study to Assess the Incidence of Hemolysis, Safety, and Efficacy of Tafenoquine (SB-252263, WR238605) versus Primaquine in the Treatment of Subjects with *Plasmodium vivax* Malaria.  **Brief description:** drug(s)/intervention(s): Tafenoquine; Primaquine; design/arms: double-blind, randomized. |
| NCT02509481  **Countries:** Burkina Faso | **Title:** Repeat Ivermectin Mass Drug Administrations for Control of Malaria: A Pilot Safety and Efficacy Study  Brief description: Phase 2; design/arms: cluster-randomized, randomized. |
| NCT03241901  **Countries:** Tanzania | **Title:** Aiming at Prolonging the Therapeutic Life Span of Artemisinin-Based Combination Therapies in an Era of Imminent *Plasmodium falciparum* Resistance in Bagamoyo District, Tanzania – New Strategies with Old Tools  Brief description: Phase 4; drug(s)/intervention(s): Artemether-Lumefantrine and Primaquine; design/arms: randomized. |
| NCT03355664  **Countries:** Cambodia, Vietnam | **Title:** A Multi-Centre, Open-Label Randomised Trial to Assess the Efficacy, Safety and Tolerability of the Triple ACT Artemether-Lumefantrine + Amodiaquine (AL+AQ) Compared to the ACT Artemether-Lumefantrine (AL) in Uncomplicated Falciparum Malaria in Cambodia and Vietnam  **Brief description:** design/arms: open-label, randomized. |
| NCT04315272  **Countries:** Burkina Faso | **Title:** Gut and Azithromycin Mechanisms in Infants and Neonates in Burkina Faso  Brief description: design/arms: double-blind, placebo-controlled, randomized. |
| **TUBERCULOSIS (*n* = 12)** | |
| ACTRN12614000372684 **Countries:** Vietnam | **Title:** A Randomized Controlled Trial of Community-Based Screening for Tuberculosis (the ACT 3 Study)  **Brief description:** design/arms: cluster-randomized, randomized. |
| NCT00495651  **Countries:** Côte d'Ivoire | **Title:** Benefits and Risks of Early Antiretroviral Treatment and Six-Month Isoniazid Prophylaxis in HIV-Infected Adults in Africa  **Brief description:** Phase 3; drug(s)/intervention(s): Isoniazid; design/arms: randomized. |
| NCT01755598  **Countries:** Kenya, South Africa, Zambia | **Title:** A Phase IIb, Double-Blind, Randomised, Placebo-Controlled Study to Evaluate the Efficacy, Safety and Immunogenicity of GSK Biologicals’ Candidate Tuberculosis (TB) Vaccine GSK 692342 Against TB Disease, in Adults Aged 18–50 Years, Living in a TB Endemic Region  **Brief description:** Phase 2; design/arms: double-blind, placebo-controlled, randomized. |
| NCT01865487  **Countries:** South Africa | **Title:** A Phase I/IIa Double-Blind, Randomized, Placebo-Controlled, Dose-Finding Study to Evaluate the Safety and Immunogenicity of AERAS-456 in HIV-negative Adults with and without Latent Tuberculosis Infection  **Brief description:** Phase 1; drug(s)/intervention(s): AERAS-456; design/arms: open-label, double-blind, placebo-controlled, randomized. |
| NCT01924286  **Countries:** South Africa | **Title:** Preventing Tuberculosis-Associated Immune Reconstitution Inflammatory Syndrome in High-Risk Patients: A Randomized Placebo-Controlled Trial of Prednisone (Pred-ART Trial)  **Brief description:** drug(s)/intervention(s): Prednisone; Antiretroviral therapy; design/arms: open-label, double-blind, placebo-controlled, randomized. |
| NCT01927159  **Countries:** South Africa | **Title:** A Phase I/II Trial of the Pharmacokinetics, Tolerability, and Safety of Once-Weekly Rifapentine and Isoniazid in HIV-1-infected and HIV-1-uninfected Pregnant and Postpartum Women with Latent Tuberculosis Infection  **Brief description:** Phase 1B; drug(s)/intervention(s): ID93 + GLA-SE; design/arms: double-blind, placebo-controlled, randomized. |
| NCT02075203  **Countries:** South Africa | **Title:** A Randomized, Placebo Controlled, Partially Blinded Phase II Study to Evaluate Safety, Immunogenicity, and Prevention of Infection with *Mycobacterium tuberculosis* of AERAS-404 and BCG Revaccination in Healthy Adolescents  **Brief description:** Phase 2; drug(s)/intervention(s): AERAS-404; design/arms: placebo-controlled, randomized. |
| NCT02409290  **Countries:** Ethiopia, Georgia, India, Moldova, South Africa, Uganda | **Title:** STREAM: A Randomized Non-Inferiority Trial to Evaluate a Short Standardized Regimen for the Treatment of Rifampicin-Resistant Tuberculosis  **Brief description:** drug(s)/intervention(s): Rifampin; design/arms: non-inferiority, randomized. |
| NCT02410772  **Countries:** Brazil, China, Haiti, India, Kenya, Malawi, Peru, South Africa, Thailand, Uganda, United States, Vietnam, Zimbabwe | **Title:** Rifapentine-containing treatment shortening regimens for pulmonary tuberculosis: A randomized, open-label, controlled phase 3 clinical trial  **Brief description:** Phase 3; design/arms: open-label, non-inferiority, randomized. |
| NCT02651259  **Countries:** Haiti, Kenya, Malawi, Thailand, Zimbabwe | **Title:** A Phase I/II Trial of the Pharmacokinetics, Tolerability, and Safety of Once-Weekly Rifapentine and Isoniazid in HIV-1-infected and HIV-1-uninfected Pregnant and Postpartum Women with Latent Tuberculosis Infection  **Brief description:** Phase 1; drug(s)/intervention(s): Rifapentine; Isoniazid; Twelve directly observed once-weekly doses of RPT; design/arms: open-label. |
| NCT03086486  **Countries:** Georgia, Moldova, Russia, South Africa | **Title:** A Phase 3 Partially-Blinded, Randomized Trial Assessing the Safety and Efficacy of Various Doses and Treatment Durations of Linezolid Plus Bedaquiline and Pretomanid in Participants with Pulmonary Infection of Either Extensively Drug-Resistant Tuberculosis (XDR-TB), Pre-XDR-TB or Treatment Intolerant or Non-Responsive Multi-Drug Resistant Tuberculosis (MDR-TB)  **Brief description:** Phase 3; drug(s)/intervention(s): Linezolid; design/arms: double-blind, randomized. |
| NCT04734652  **Countries:** South Africa | **Title:** INSTI’s for the Management of HIV-Associated TB (INSIGHT Study): A Phase 2b Study to Evaluate the Efficacy, Safety and Pharmacokinetics of a Combination of Bictegravir, Emtricitabine, and Tenofovir Alafenamide Fumarate for Treatment of HIV-1 Infection in Patients with Drug-Susceptible Tuberculosis on a Rifampicin-Based Treatment Regimen: A Phase 2b Open-label Randomised-Controlled Trial  **Brief description:** Phase 2B; drug(s)/intervention(s): Bictegravir; Emtricitabine; Tenofovir; Rifampicin; design/arms: open-label, randomized. |
| **NEGLECTED TROPICAL DISEASES (*n* = 7)** | |
| NCT00123916  **Countries:** Argentina, Bolivia, Brazil, Colombia, El Salvador | **Title:** Benznidazole Evaluation For Interrupting Trypanosomiasis (BENEFIT) Trial: A Randomized Double-Blind Placebo-Controlled Clinical Trial of Benznidazole in Patients with Chronic Chagas’ Heart Disease  **Brief description:** Phase 40; drug(s)/intervention(s): Benznidazole; design/arms: double-blind, placebo-controlled, randomized. |
| NCT01284855  **Countries:** Nepal | **Title:** A Randomized, Double-Blind, Clinical Trial of Two Dose Regimens of VINS Polyvalent Antivenom for the Treatment of Snake Bites with Neurotoxic Envenoming in Nepal  **Brief description:** Phase 2; design/arms: double-blind, randomized. |
| NCT01955252  **Countries:** Papua New Guinea | **Title:** Yaws Elimination on Lihir Island, New Ireland Province, PNG: A Proof of Principle of Mass Drug Treatment with Azithromycin.  **Brief description:** drug(s)/intervention(s): Azithromycin; design/arms: non-inferiority, randomized. |
| NCT02184689  **Countries:** Democratic Republic of the Congo | **Title:** Efficacy and Safety of Fexinidazole in Children at Least 6 Years of Age and Weighing Over 20 kg with Human African Trypanosomiasis (HAT): a Prospective, Multicentre, Open-Label Study, Plug-in to the Pivotal Study  **Brief description:** Phase 2; design/arms: open-label, randomized. |
| NCT02899936  **Countries:** Fiji, Sri Lanka, Haiti, India, Indonesia, Papua New Guinea | **Title:** Community Based Safety and Efficacy Study of 2-drug (Diethylcarbamazine and Albendazole) versus 3-drug (Ivermectin, Diethylcarbamazine and Albendazole) Therapy for Lymphatic Filariasis in Indonesia.  **Brief description:** drug(s)/intervention(s): Albendazole; Ivermectin. |
| NCT03245398  **Countries:** Tanzania | **Title:** Efficacy and Safety of a Single-Dose Regimen and a Multi-Dose Regimen of Mebendazole Against Hookworm Infections in Children: A Randomized Controlled Trial  **Brief description:** Phase 4; design/arms: double-blind, randomized. |
| NCT04188301  **Countries:** Ghana | **Title:** Safety and Efficacy of Combination Therapy with Ivermectin, Diethylcarbamazine, and Albendazole (IDA) for Individuals with Onchocerciasis.  **Brief description**: drug(s)/intervention(s): Ivermectin; Albendazole; design/arms: open-label, randomized. |
